# Supplementary material for: Efficient DNA fluorescence labeling via base excision trapping
Source: Nat Commun. 2022 Aug 26;13:5043. doi: 10.1038/s41467-022-32494-8 (PMC9418136; doi:10.1038/s41467-022-32494-8)
Supplement: Supplementary file 1 — Supplementary Information [file 41467_2022_32494_MOESM1_ESM.pdf]

Efficient DNA fluorescence labeling via base excision trapping

Yong Woong Jun,[1] Emily M. Harcourt,[2] Lu Xiao,[1] David L. Wilson,[1] and Eric T. Kool[1*]

[1]*Department of Chemistry, ChEM-H Institute, and Stanford Cancer Institute, Stanford University, Stanford, CA, 94305, USA*
[2]*Department of Chemistry, Le Moyne College, Syracuse, NY, 13214, USA*

[*]To whom correspondence should be addressed: kool@stanford.edu

Table of Contents

Experimental Procedures 3
Instrumentation 3
Chemicals and Enzymes 3
Synthesis of UBER Red 3
Supplementary Scheme 4
Supplementary Tables 4
Supplementary Table 1 4
Supplementary Table 2 5
Supplementary Figures 6
Supplementary Figure 1 6
Supplementary Figure 2. 7
Supplementary Figure 3. 7
Supplementary Figure 4 7
Supplementary Figure 5. 8
Supplementary Figure 6. 8
Supplementary Figure 7. 9
Supplementary Figure 8 9
Supplementary Figure 9. 10
Supplementary Figure 10. 10
Supplementary Figure 11. 10
Supplementary Figure 12. 11
Supplementary Figure 13 11
Supplementary Figure 14. 12
Supplementary Figure 15. 12
Supplementary Figure 16. 13
Supplementary Figure 17. 13
Supplementary Figure 18 13
Supplementary Figure 19 14
Supplementary Figure 20. 14
Supplementary Figure 21. 14
Uncropped gels presented in supplementary figures 15
References 15

Experimental Procedures

Instrumentation

Fluorescence images were recorded on an inverted Zeiss LSM 780 multiphoton laser scanning confocal microscope in cell sciences imaging facility (CSIF) at Stanford. Fluorescence emission and excitation spectra were recorded on a Jobin Yvon-Spex Fluorolog 3 spectrometer with an external temperature controller, or a Fluoroskan Ascent microplate fluorometer, or a Bio-Tek Synergy HT microplate fluorimeter. Typhoon gel imager, GE Healthcare.

Chemicals and Enzymes

All chemicals were purchased from Sigma Aldrich, Thermo Fisher, and Combi-blocks and used without further purification. UBER green was synthesized as described previously, or was purchased from Skunkworks Biosciences (Palo Alto, CA).[1] Uracil-DNA glycosylase (UDG), N-methylpurine DNA glycosylase (MPG), Klenow fragment (exo-), Therminator, Bst 3.0, T7, Protocript II, Taq, and phi29, were purchased from New England Biolab (NEB). RevertAid H Minus Reverse Transcriptase and Ribolock RNase Inhibiotor were purchased from ThermoFisher. Primaty antibody Anti-G3BP-1 (SAB4500043-100 ug) was purchased from Sigma-Aldrich. oYo-Link oligo custom for antibody oligonucleotide conjugation was purchased from Alpha Thera.[2] Easy-DNA gDNA purification kit was purchased from ThermoFisher (Invitrogen, K180001). Nick translation DNA labeling system 2.0 (ENZ-Gen111-0050) and fluorescein-dUTP (SEEBRIGHT Green 496 dUTP) were purchased from Enzo Life Science. 3k Centrifugal filters were purchased from Amicon.

Synthesis of UBER Red

Synthesis of *tert*-butyl-(2-bromoethoxy)carbamate (4). This compound was prepared as described by Javorskis and cowerkers.[3] *Tert*-butyl hydroxycarbamate (1.0 g, 7.51 mmol) and 1,8-diazabicyclo[5.4.0]undec-7-ene (DBU, 1.14 g, 7.51) were dissolved in 1,2-diboromoethane (5 mL), and stirred overnight. The reaction was then taken into dichloromethane, washed with 1M HCl and saturated brine, dried over anhydrous sodium sulfate, and concentrated *in vacuo*. The resulting crude was purified by flash column chromatography (ethyl acetate:hexane = 1:20). Yield 52%.

Synthesis of (*E*)-*N,N*-dimethyl-4-(2-(pyridine-4-yl)vinyl)aniline (3). 4-(dimethylamino)benzaldehyde (1.5 g, 10 mmol), 4-methylpyridine (930 mg, 10 mmol), and potassium butoxide (1.34 g, 12 mmol) were dissolved in anhydrous DMF (50 mL), and stirred for 2 h at 80°C. After cooling it down to rt, the reaction was taken into dichloromethane (200 mL), washed with water (200 mL), 1 M KOH (200 mL), and saturated brine, dried over anhydrous sodium sulfate, and concentrated *in vacuo*. The resulting crude was purified by precipitation with dichloromethane and hexane. Yield: 87%. ^{1}H NMR (400 MHz, Chloroform-*d*): δ = 8.49 ppm (d, J = 4 Hz, 2H), 7.42 (d, J = 8 Hz, 2H), 7.30 (d, J = 4 Hz, 2H), 7.24 (s, 1H), 6.78 (d, J = 16 Hz, 1H), 6.67 (d, J = 8 Hz, 2H), 2.99 (s, 6H).

Synthesis of (*E*)-1-(2-(((*tert*-butoxycarbony)amino)oxy)ethyl)-4-(4-(dimethylamino)styryl)pyridine-1-ium (2). In an argon-flushed and flame-dried flask, **3** (105 mg, 0.468 mmol) and **4** (225 mg, 0.937 mmol) were dissolved in anhydrous acetonitrile (5 mL), and stirred for 24 h at 80°C. The reaction was concentration *in vacuo*, then purified with preparative TLC (eluent: 5% methanol and 1% trietylamine in dichloromethane). Yield = 67%. ^{1}H NMR (400 MHz, Chloroform-*d*): δ = 9.27 ppm (d, J = 8 Hz, 2H), 8.92 (s, 1H), 7.79 (d, J = 4 Hz, 2H), 7.52 (d, J = 12 Hz, 2H), 6.85 (d, J = 20 Hz 1H), 6.70 (d, J = 12 Hz, 2H). 5.03 (t, J = 6 Hz, 2H), 4.39 (t, J = 6 Hz, 2H), 3.09 (s, 6H), 1,47 (s, 9H).

Synthesis of (E)-1-(2-(ammonioxy)ethyl)-4-(4-(dimethylamino)styryl)pyridine-1-ium (1). To **2** (100 mg) dissolved in anhydrous dichloromethane (3 mL), trifluoroacetic acid (3 mL) was added dropwise. The reaction was then concentrated *in vacuo*, then the product was purified with HPLC. Purification yield = 43%. ^{1}H NMR (400 MHz, Chloroform-*d*): δ = 8.5 ppm (m, 2H), 7.95 (d, J = 4 Hz, 2H), 7.83 (d, J = 16 Hz, 1H), 7.59 (d, J = 8 Hz, 2H), 7.07 (d, J = 16 Hz, 1 H), 6.77 (d, J = 8 Hz, 2H), 4.70 (t, J = 4 Hz, 2H), 4.49 (t, J = 4 Hz, 2H), 3.05 (s, 6H). ESI-MS [M+H]: Calculated: 285.39; Observed: 285.7.

Supplementary Scheme

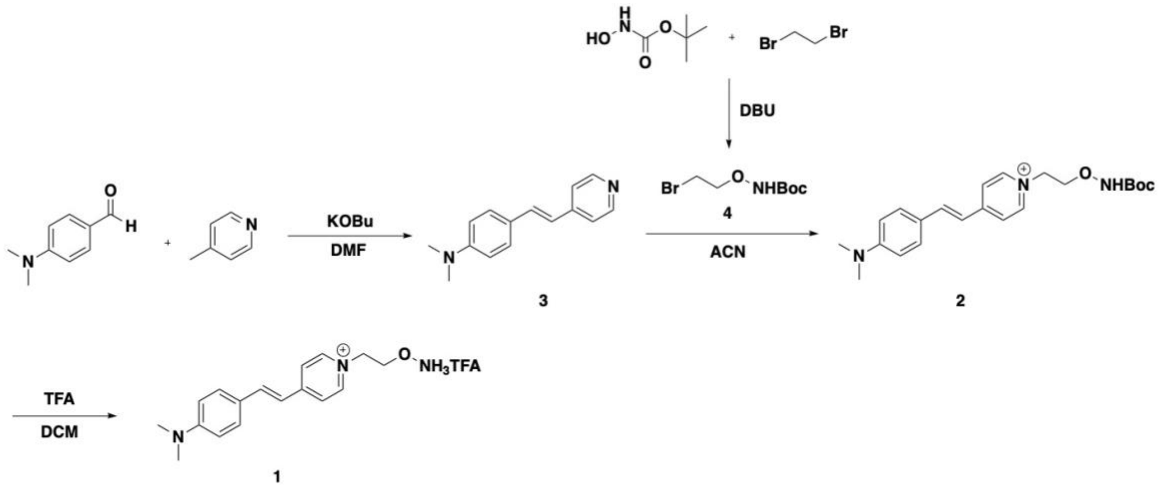

Scheme 1. Synthetic route of UBER Red. Compound **3** was synthesized through condensation reaction, which was used for the substitution reaction with compound **4** to synthesize compound **2**. UBER Red (compound **1**) was synthesized through the boc-deprotection step from compound **2**.

Supplementary Tables

Supplementary Table 1. Comparison of different DNA labeling methods.
MTase; methyltransferases, TGT; tRNA guanine transglycosylase, β-GT; β-glucosyltransferase.
(note that cost will vary with scale and commercial source; see Suppl. Fig. S6)

	**BETr**	Phosphoramidite		Triphosphate		MTase	TGT	β-GT
		Fluorophore	Click handle	Fluorophore	Click handle			
Labeling long DNA	✓	✗	✗	✓	✓	✓	✓	✓
One-step labeling	✓	✓	✗	✓	✗	✓	✓	✓
In situ synthesis & labeling	✓	✓	✗	✓	✗	✗	✗	✗
Light-up	✓	✗	△ (Rare)	✗	△ (Rare)	✗	✗	✗
Turnover labeling	✓	✗	✗	✗	✗	Possibly	✗	✗
Cost efficiency	High	Expensive modified phosphoramidites		Expensive modified triphosphates		Very expensive modified cofactors		
Enzyme compatibility	High	n/a	n/a	Low	High	High	High	High
Reagent availability	Commercially available					Synthesis required for cofactors		
Labeling site specificity	△ only with synthetic template	High		Low	△ only with synthetic template	High	High	High
Consecutive Multi-labeling	✓	✓	✓	✗	✓	✗	✗	✗
Requirement for synthetic chemistry lab	No	Yes	Yes	No	Yes	No	No	No

Supplementary Table 2. List of oligonucleotides used in the experiments. Key nucleobases are marked in bold.

#	Source	Name	Sequence (5’ → 3’)
1	IDT	dU-sample	GACGC**U**GAGACGAAGTCTCCGCGTC
2	IDT	2 dA	TCGCCTGGT**A**CTGCCGCTCGCGTCCGTC**A**TCGGCGTCTGTGGCCGCCATGCCGTAGCCAG
3	IDT	5 dA	TCGC**A**TGGTTCT**A**CCGCTCG**A**GTCCGTC**A**TCGGCGTC**A**GTGGCCGCCATGCCGTAGCCAG
4	IDT	9 dA<br>(Target DNA)	TCGC**A**TGGT**A**CTT**A**CGCT**A**GG**A**TCC**A**TCGT**A**GGCG**A**CCG**A**GGCCGCCATGCCGTAGCCAG
5	IDT	11 dA	TCGC**A**TG**A**TT**A**CT**A**CCG**A**TCG**A**GTC**A**GTC**A**TCG**A**CG**A**CCG**A**GGCCGCCATGCCGTAGCCAG
6	IDT	Primer	CTGGCTACGGCATGGCGG
7	IDT	U	CTGGCTACG**U**CATGGCGG
8	IDT	UU	CTGGCTACG**UU**ATGGCGG
9	IDT	UUU	CTGGCTAC**UUU**ATGGCGG
10	IDT	UGU	CTGGCTAC**U**G**U**ATGGCGG
11	IDT	UGGU	CTGGCTA**U**GG**U**ATGGCGG
12	IDT	cDNA	CCGCCATGCCGTAGCCAG
13	IDT	1A	TCGC**A**TGGT**A**CTT**A**CGCTTGGCTCCGTCGTTGGCGCCCGGG**A**CCGCCATGCCGTAGCCAG
14	IDT	2A	TCGC**A**TGGT**A**CTT**A**CGCTTGGCTCCGTCGTTGGCGCCCGG**AA**CCGCCATGCCGTAGCCAG
15	IDT	3A	TCGC**A**TGGT**A**CTT**A**CGCTTGGCTCCGTCGTTGGCGCCCG**AAA**CCGCCATGCCGTAGCCAG
16	IDT	4A	TCGC**A**TGGT**A**CTT**A**CGCTTGGCTCCGTCGTTGGCGCCC**AAAA**CCGCCATGCCGTAGCCAG
17	IDT	5A	TCGC**A**TGGT**A**CTT**A**CGCTTGGCTCCGTCGTTGGCGCC**AAAAA**CCGCCATGCCGTAGCCAG
18	PAN	END	CTGGCTACGGCATGGCG**U**
19	IDT	END +1	CTGGCTACGGCATGGC**U**G
20	IDT	END +2	CTGGCTACGGCATGG**U**GG
21	IDT	END +3	CTGGCTACGGCATG**U**CGG
22	IDT	END +4	CTGGCTACGGCAT**U**GCGG
23	IDT	dU/dI	GCGAGCTAAAGCGTCGACGGAACGTCG**I**CGCGTTA**U**CTCGC
24	IDT	RCA Primer	CAGGAAACAGCTATGACC
25	IDT	ssDNA-1	ACATTCCT**U**AGTCTGAAACATTACAGCTTGCTACACGAGAAGAGCCGCCATAGTA
26	IDT	ssDNA-2	TCAACTGCCTGGTGATAAAACGACACT**U**CGTGGGAATCTACTATG**U**CGGCTCTTC
27	IDT	ssDNA-3	TATCACCA**U**GCAGTTGACAGTGTAGC**U**AGCTGTAATAGATGCGAG**U**GTCCAATAC
28	IDT	ssDNA-linker	TTCAGACTTAGGAATGTGCTTCCCACGTAGTGTCGTTTGTATTGGACCCTCGCATTTACTGGCCGTCGTTTTAC
29	Alpha Thera	Tethering oligo	(BPA-Protein G)-GTAAAACGACGGCCAGT[4]
30	IDT	7 mer	GAC**I**CGA
31	IDT	9 mer	TGAC**I**CGAC
32	IDT	11 mer	CTGAC**I**CGACC
33	IDT	Turnover Template dA	TGGTCG**A**GTCAGT
34	IDT	Turnover Template dT	TGGTCG**T**GTCAGT
35	IDT	Turnover Template dG	TGGTCG**G**GTCAGT

36	IDT	Turnover Template dC	TGGTCG**C**GTCAGT
37	IDT	Spectator DNA 1	GAACCGCTTGGCCACGGCCTCGCCCACCTCCTTGCTCAGGG
38	IDT	Spectator DNA 2	GATCAGGATGGCGCTGGTGGTCTCGGTCAGGCCGTAGCCCTGCCGGATG CCGGGCAGGTG
39	IDT	Spectator DNA 3	GGCCTCGAAGAAGGGCACCACCTTGCCCACGGCGCCGGGCTTGTCGTCG CCCTCGGGGGT
40	IDT	Spectator DNA 4	GCACAGCTCGCCCCGCTGGTTCACGCCCAGGGTCTTGCCGGTGTCCAGG TCCACCACCTT
41	IDT	Spectator DNA 5	CAGGGCGTTGGTGGCCTCGGGGTTGTTCACGTAGCCGCTCATGATCATG GGGCCCCGCAC
42	IDT	TDN-fluorescein	TTCAGACTTAGGAATGTGCTTCCCACG/**Fl**/AGTGTCGTTTGTATTGGACCC TCGCAT
43	IDT	Mismatch α	TGGTC**T**TGTCAGT
44	IDT	Mismatch β	TGGT**A**GTGTCAGT
45	IDT	Mismatch γ	TGG**G**CGTGTCAGT
46	IDT	Mismatch δ	TG**T**TCGTGTCAGT
47	IDT	DNA_dU	AGGGTGTGGTC**U**GCCTAGAA
48	IDT	DNA	TCGACGCAGCTTCTAGGCAGACCACACCCT
49	IDT	RNA	ucgacgcagcuucuaggcagaccacacccu
50	IDT	RNA template	cccauuuacccauuuacucccacacgcccu
51	IDT	RT primer 1	AGGGTGTGGTCTG
52	IDT	RT primer 2	AGGGCGTGTG
53	IDT	4dU	TCG CCT **U**GT ACT G**U**C GCT C**U**C GTC CG**U** CAT CGG
54	IDT	4dU_cDNA	CCG ATG CCG GAC GCG AGC GCC AGT ACC AGG CGA
55	IDT	Cons_3dU	CTA ATG **UUU** CGA CGG AAC GTC GAC GCG TTA G
56	IDT	Long Target DNA	TCGCATGGTACTTACGCTTGGCTCCGTCGTTGG
57	IDT	3bp	CGG**I**GCC**I**AGC
58	IDT	4bp	GCCA**I**GCGT**I**AGTA
59	IDT	5bp	GAGCC**I**AGCGT**I**AGTAC
60	IDT	Target RNA	acuggucgugtcaguac

Supplementary Figures

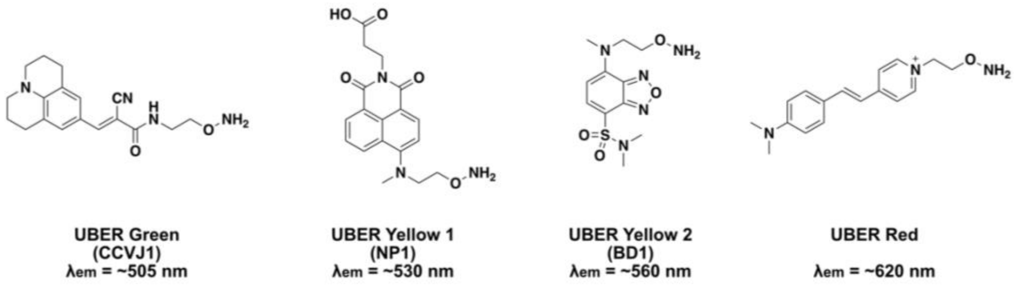

Supplementary Figure 1. Structures of UBER reactive dyes developed recently[1] All are designed to have low fluorescence in solution due to rapid bond rotations, but to yield a light-up signal in DNA due to rigidification. They contain short tethers to an aminooxy group that reacts with abasic deoxyribose generated during base excision repair. UBER Green, UBER Yellow 1 and 2 were reported previously;[1] UBER Red was synthesized here. UBER Green (CCVJ-1) can be obtained from Skunkworks Biosciences (skunkworksbiosciences.com).

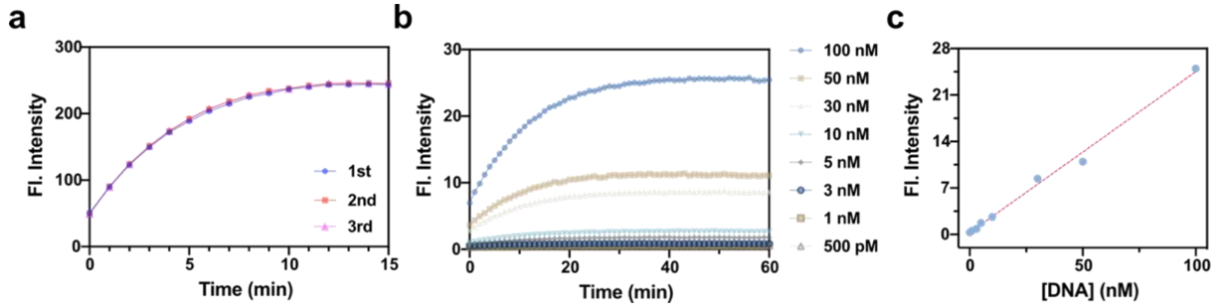

Supplementary Figure 2. Reproducibility of the method (a) Triplicate fluorescence enhancements of 10 µM dU-sample incubated with 20 U/mL UDG and 10 µM UBER at 37 ℃ from distinct samplesshowing reproducibility. (b) Fluorescence intensity changes over time with varied concentration of an oligonucleotide (**dU_sample**) containing one dU upon the addition of 10 µM CCVJ-1 and 20 U/mL UDG at 37 ℃. (c) Fluorescence intensities comparison at 60 min depending on the concentration of **dU-sample**. The fluorescence was measured on a Fluoroskan (485 nm/538 nm).

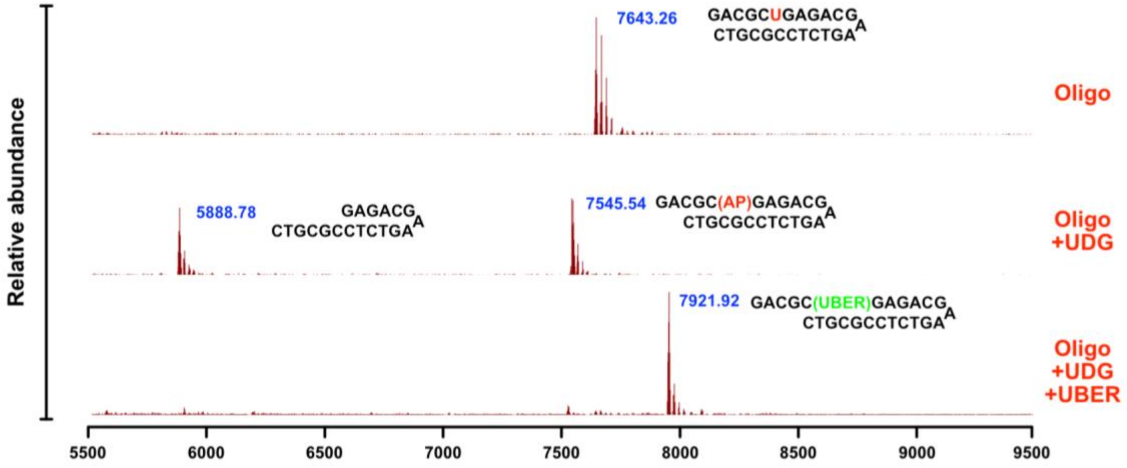

Supplementary Figure 3. MALDI-TOF analysis data showing covalent linkage with UBER The dU-sample ODN (2 µM) was incubated with 20 U/mL UDG and in the presence and absence of 10 µM of UBER overnight at 37 ℃. Relative percentage of peaks were calculated based on the sum of the heights of the peaks.

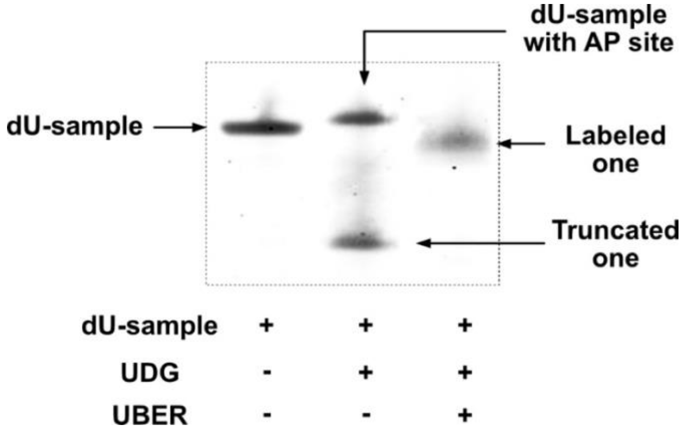

Supplementary Figure 4. Denaturing PAGE gel electrophoresis analysis of dU-containing oligonucleotide labeling, showing stabilization of AP site in the presence of UBER reagent. (a) dU-sample (2 μM) was incubated with 20 U/mL UDG for 24 h in the presence and absence of UBER (10 μM). The gel was visualized with SYBR gold.

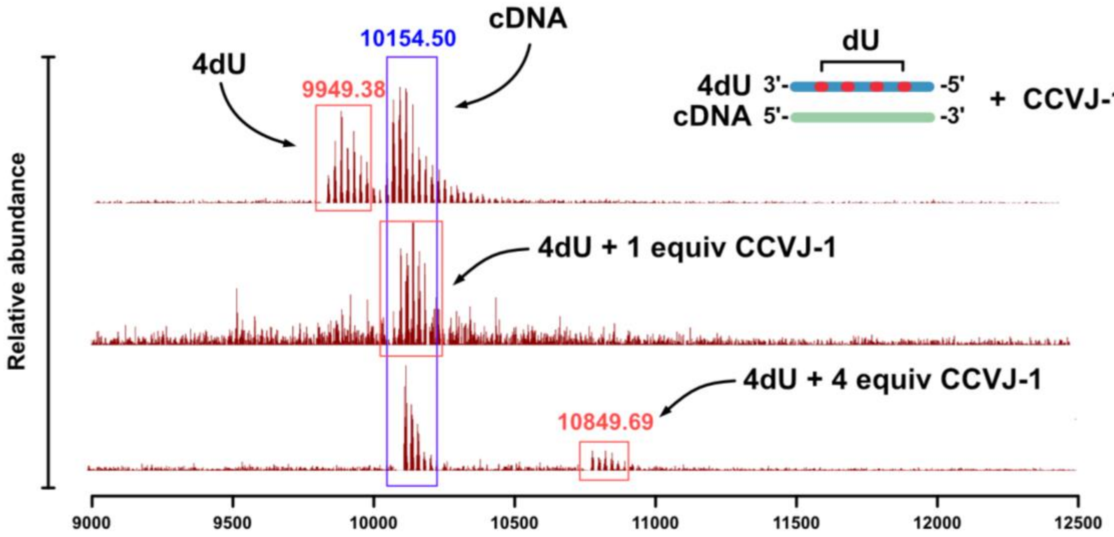

Supplementary Figure 5. Documentation of multi-labeling of DNA 4dU (10 µM) hybridized with 4dU_cDNA (11 µM) was labeled with 10 µM or 100 µM CCVJ-1 and 2 U/mL UDG for 24 h, then subjected to ethanol precipitation. The pellets were dissolved in water for MALDI analysis.

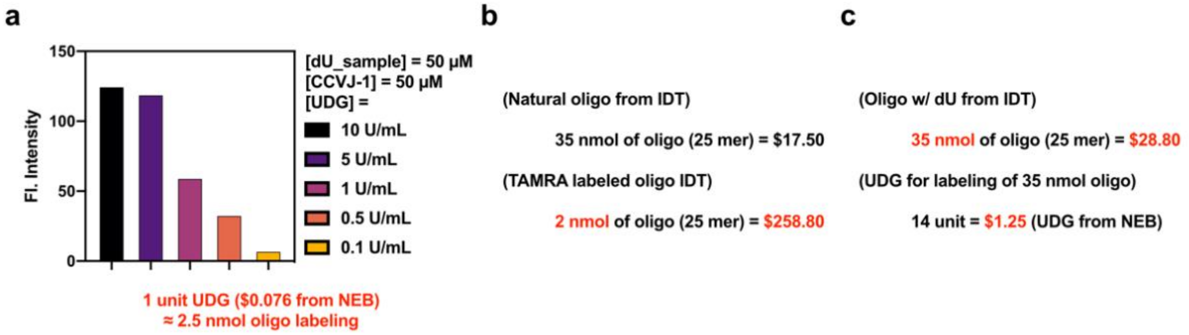

Supplementary Figure 6. Enzyme optimization and cost estimates (a) Titration of the amount of UDG for labeling 50 µM **dU_sample** with 50 µM CCVJ-1 at 37 °C for 60 min, showing that 1 unit of UDG labels approximately 2.5 nmol of **dU_sample**. (b) Cost of a commercial TAMRA-labeled oligonucleotide (25-mer), which costs about $258.80 for 2 nmol from IDT. (c) Cost of a commercial DNA oligo (25-mer) containing a single dU and enzyme (UDG from NEB) required for the labeling. The total cost is less than $1 per nmol of UBER-labeled DNA. (Note that costs may vary with supplier and scale. Purification costs are also not included here; HPLC or PAGE purification add further costs for synthetic ODNs).

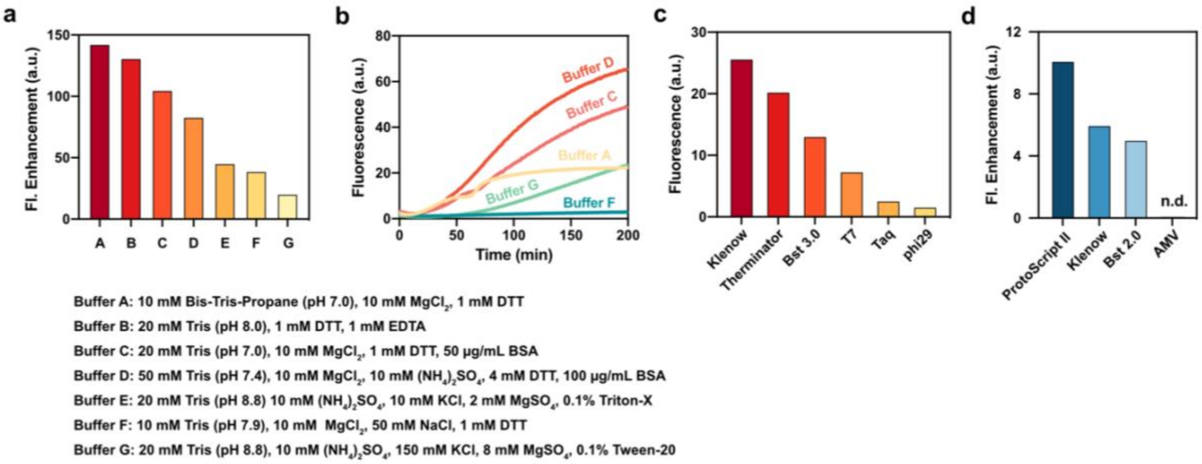

Supplementary Figure 7. Screening of buffers (a) BETr efficiency measured with 10 µM **dU_sample**, 10 µM CCVJ-1, 20 U/mL UDG in different compositions of buffers. (b) *in situ* DNA synthesis and BETr observed in different buffers with 1 µM **9dA**, 5 µM primer, [dNTP (A, G, and C)] = 50 µM, [dUTP] = 100 µM, [Klenow] = 2 U/mL. (c) The efficiency of *in situ* synthesis and BETr labeling measured with different polymerases in the buffer provided with the polymerases. (d) The efficiency of *in situ* synthesis and BETr labeling on RNA template measured with **RNA template** (0.5 µM), **RT primer 2** (1 µM), and different reverse transcriptases in 50 mM tris buffer pH 7.4 with 50 mM NaCl and 5 mM $MgCl_2$. The fluorescence was measured on a Fluoroskan Ascent microplate reader (485 nm/538 nm) at 37 °C. The bar graphs were plotted with the data at 5 h of incubation.

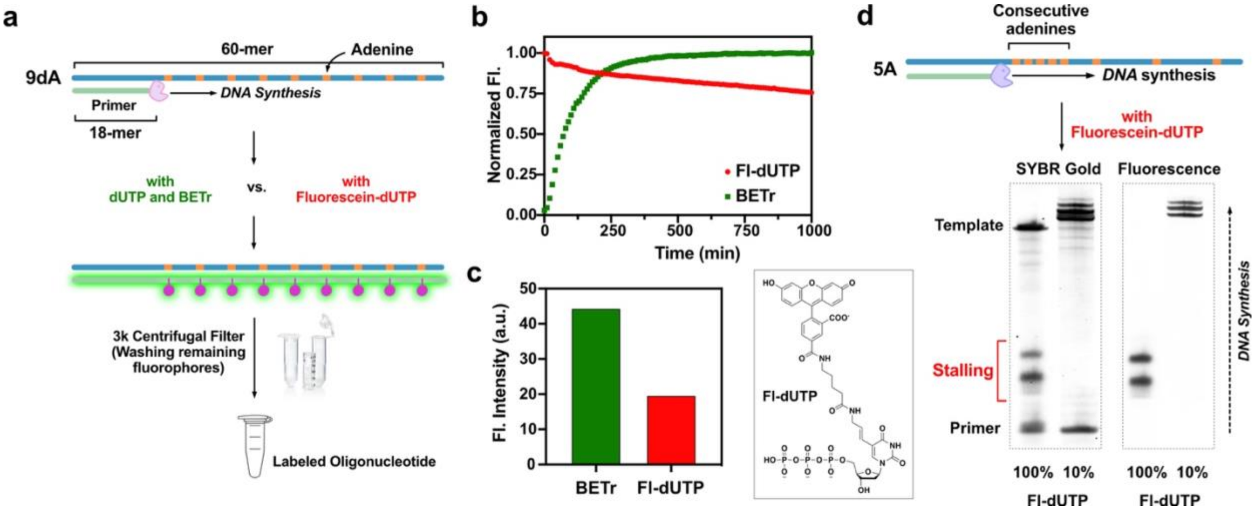

Supplementary Figure 8. Comparison of BETr with a conventional polymerase-mediated labeling method with Fl-dUTP (a) Illustration of *in situ* synthesis and labeling with BETr and Fl-dUTP, and following washing step for the measurement of brightness. (b) Normalized fluorescence change while each probe gets incorporated into newly synthesized oligonucleotides. (c) Fluorescence intensities of the oligonucleotides labeled with BETr or Fl-dUTP, after the filtration step removing remaining unreacted dyes and the chemical structure of Fl-dUTP. Note that relative intensities will likely be dependent on dye choice for comparison, as dyes brighter than fluorescein are known. (d) 15% Denaturing PAGE gel electrophoresis after the DNA synthesis with 5A and primer under fluorescence imaging setup with FITC channel. Considering that labeling with Fl-dUTP stalls at consecutive adenine sites, labeling gDNA with Fl-dUTP through nick translation requires the dilution of the Fl-dUTP with dTTP which reduce the labeling frequency. The fluorescence was measured on a Fluoroskan Ascent microplate reader (485 nm/538 nm). [9dA] = 1 μM, [5A] = 1 μM, [Primer] = 5 μM, dNTP(A,C,G) = 100 μM, [dUTP] = 100 μM, [Fl-dUTP] = 10 μM, [UBER] = 10 μM, UDG = 20 U/ml, [Klenow] = 2 U/mL.

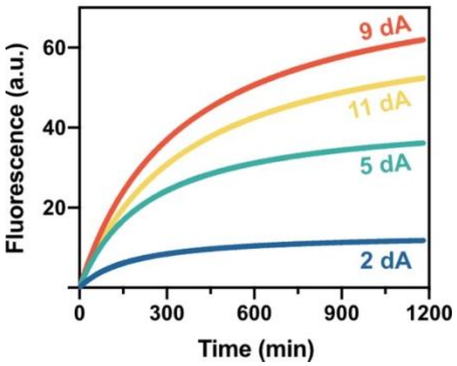

Supplementary Figure 9. Fluorescence enhancement depending on the number of dA on template Fluorescence enhancement depending on the number of labeling sites in ODNs with [CCVJ-1] = 20 μM, [UDG] = 10 U/mL, [Templates] = 1 μM, [Primer] = 5 μM, [dATP, dCTP, dGTP] = 50 μM, [dUTP] = 100 μM, [Polymerase] = 2 U/mL, measured in a Fluoroskan Ascent microplate reader (485/528 nm)

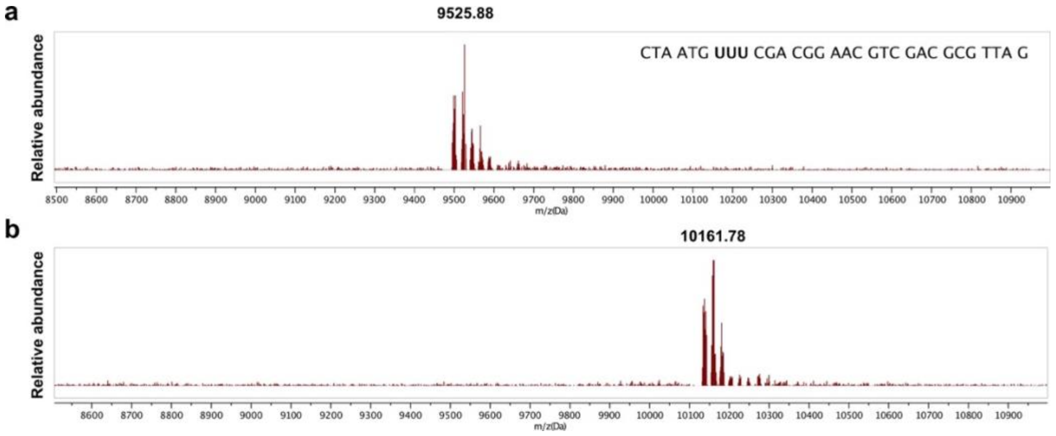

Supplementary Figure 10. Documentation of consecutive labeling of DNA with UDG Cons_3dU (10 µM) was labeled with 100 µM CCVJ-1 and 2 U/mL UDG for 24 h, then subjected to ethanol precipitation. The pellets were dissolved in water for MALDI analysis.

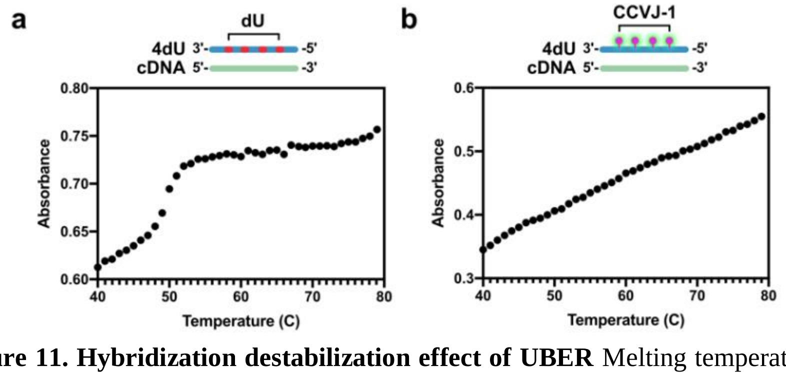

Supplementary Figure 11. Hybridization destabilization effect of UBER Melting temperature of unlabeled 4dU dsDNA (10 µM) and fully labeled 4dU dsDNA (10 µM) with CCVJ-1, implying that 4dU fully labeled with CCVJ-1 is no longer hybridized with cDNA.

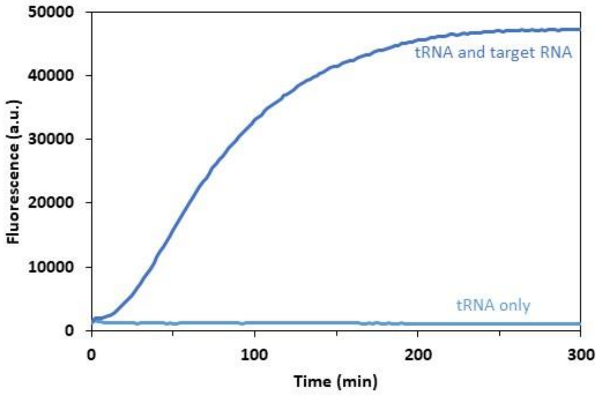

Supplementary Figure 12. Sequence specificity test with RNA. Fluorescence changes due to DNA synthesis and labeling in the presence of baker's yeast tRNA with and without target RNA. [CCVJ-1] = 20 µM, [**RNA**] = 0.5 µM, [**RT Primer 1**] = 1 µM, [tRNA] = 100 µg/mL. 0.4 U/uL of RiboLock RNase inhibitor was included. The fluorescence was measured on a Bio-Tek Synergy HT microplate reader at 37 °C.

AATGCTACTACTATTAGTAGAATTGATGCCACCTTTTCAGCTCGCGCCCCAAATGAAAATATAGCTAAACAGGTTATTGACCATTTGCGAAATGTATCTAATGGTCAAACTAAATCTACTCGTTCGCAGAATTGGGA
ATCAACTGTTATATGGAATGAAACTTCCAGACACCGTACTTTAGTTGCATATTTAAAACATGTTGAGCTACAGCATTATATTCAGCAATTAAGCTCTAAGCCATCCGCAAAAATGACCTCTTATCAAAAGGAGCAAT
TAAAGGTACTCTCTAATCCTGACCTGTTGGAGTTTGCTTCCGGTCTGGTTCGCTTTGAAGCTCGAATTAAAACGCGATATTTGAAGTCTTTCGGGCTTCCTCTTAATCTTTTTGATGCAATCCGCTTTGCTTCTGACTAT
AATAGTCAGGGTAAAGACCTGATTTTTGATTTATGGTCATTCTCGTTTTCTGAACTGTTTAAAGCATTTGAGGGGGATTCAATGAATATTTATGACGATTCCGCAGTATTGGACGCTATCCAGTCTAAACATTTTACTA
TTACCCCCTCTGGCAAAACTTCTTTTGCAAAAGCCTCTCGCTATTTTGGTTTTTATCGTCGTCTGGTAAACGAGGGTTATGATAGTGTTGCTCTTACTATGCCTCGTAATTCCTTTTGGCGTTATGTATCTGCATTAGTT
GAATGTGGTATTCCTAAATCTCAACTGATGAATCTTTCTACCTGTAATAATGTTGTTCCGTTAGTTCGTTTTATTAACGTAGATTTTTCTTCCCAACGTCCTGACTGGTATAATGAGCCAGTTCTTAAAATCGCATAAG
GTAATTCACAATGATTAAAGTTGAAATTAAACCATCTCAAGCCCAATTTACTACTCGTTCTGGTGTTTCTCGTCAGGGCAAGCCTTATTCACTGAATGAGCAGCTTTGTTACGTTGATTTGGGTAATGAATATCCGGT
TCTTGTCAAGATTACTCTTGATGAAGGTCAGCCAGCCTATGCGCCTGGTCTGTACACCGTTCATCTGTCCTCTTTCAAAGTTGGTCAGTTCGGTTCCCTTATGATTGACCGTCTGCGCCTCGTTCCGGCTAAGTAACAT
GGAGCAGGTCGCGGATTTCGACACAATTTATCAGGCGATGATACAAATCTCCGTTGTACTTTGTTTCGCGCTTGGTATAATCGCTGGGGGTCAAAGATGAGTGTTTTAGTGTATTCTTTTGCCTCTTTCGTTTTAGGTT
GGTGCCTTCGTAGTGGCATTACGTATTTTACCCGTTTAATGGAAACTTCCTCATGAAAAAGTCTTTAGTCCTCAAAGCCTCTGTAGCCGTTGCTACCCTCGTTCCGATGCTGTCTTTCGCTGCTGAGGGTGACGATCCC
GCAAAAGCGGCCTTTAACTCCCTGCAAGCCTCAGCGACCGAATATATCGGTTATGCGTGGGCGATGGTTGTTGTCATTGTCGGCGCAACTATCGGTATCAAGCTGTTTAAGAAATTCACCTCGAAAGCAAGCTGATA
AACCGATACAATTAAAGGCTCCTTTTGGAGCCTTTTTTTTGGAGATTTTCAACGTGAAAAAATTATTATTCGCAATTCCTTTAGTTGTTCCTTTCTATTCTCACTCCGCTGAAACTGTTGAAAGTTGTTTAGCAAAATC
CCATACAGAAAATTCATTTACTAACGTCTGGAAAGACGACAAAACTTTAGATCGTTACGCTAACTATGAGGGCTGTCTGTGGAATGCTACAGGCGTTGTAGTTTGTACTGGTGACGAAACTCAGTGTTACGGTACAT
GGGTTCCTATTGGGCTTGCTATCCCTGAAAATGAGGGTGGTGGCTCTGAGGGTGGCGGTTCTGAGGGTGGCGGTTCTGAGGGTGGCGGTACTAAACCTCCTGAGTACGGTGATACACCTATTCCGGGCTATACTTAT
ATCAACCCTCTCGACGGCACTTATCCGCCTGGTACTGAGCAAAACCCCGCTAATCCTAATCCTTCTCTTGAGGAGTCTCAGCCTCTTAATACTTTCATGTTTCAGAATAATAGGTTCCGAAATAGGCAGGGGGCATTA
ACTGTTTATACGGGCACTGTTACTCAAGGCACTGACCCCGTTAAAACTTATTACCAGTACACTCCTGTATCATCAAAAGCCATGTATGACGCTTACTGGAACGGTAAATTCAGAGACTGCGCTTTCCATTCTGGCTTT
AATGAGGATTTATTTGTTTGTGAATATCAAGGCCAATCGTCTGACCTGCCTCAACCTCCTGTCAATGCTGGCGGCGGCTCTGGTGGTGGTTCTGGTGGCGGCTCTGAGGGTGGTGGCTCTGAGGGTGGCGGTTCTGAG
GGTGGCGGCTCTGAGGGAGGCGGTTCCGGTGGTGGCTCTGGTTCCGGTGATTTTGATTATGAAAAGATGGCAAACGCTAATAAGGGGGCTATGACCGAAAATGCCGATGAAAACGCGCTACAGTCTGACGCTAAAG
GCAAACTTGATTCTGTCGCTACTGATTACGGTGCTGCTATCGATGGTTTCATTGGTGACGTTTCCGGCCTTGCTAATGGTAATGGTGCTACTGGTGATTTTGCTGGCTCTAATTCCCAAATGGCTCAAGTCGGTGACGG
TGATAATTCACCTTTAATGAATAATTTCCGTCAATATTTACCTTCCCTCCCTCAATCGGTTGAATGTCGCCCTTTTGTCTTTGGCGCTGGTAAACCATATGAATTTTCTATTGATTGTGACAAAATAAACTTATTCCGTG
GTGTCTTTGCGTTTCTTTTATATGTTGCCACCTTTATGTATGTATTTTCTACGTTTGCTAACATACTGCGTAATAAGGAGTCTTAATCATGCCAGTTCTTTTGGGTATTCCGTTATTATTGCGTTTCCTCGGTTTCCTTCT
GGTAACTTTGTTCGGCTATCTGCTTACTTTTCTTAAAAAGGGCTTCGGTAAGATAGCTATTGCTATTTCATTGTTTCTTGCTCTTATTATTGGGCTTAACTCAATTCTTGTGGGTTATCTCTCTGATATTAGCGCTCAATT
ACCCTCTGACTTTGTTCAGGGTGTTCAGTTAATTCTCCCGTCTAATGCGCTTCCCTGTTTTTATGTTATTCTCTCTGTAAAGGCTGCTATTTTCATTTTTGACGTTAAACAAAAAATCGTTTCTTATTTGGATTGGGATA
AATAATATGGCTGTTTATTTTGTAACTGGCAAATTAGGCTCTGGAAAGACGCTCGTTAGCGTTGGTAAGATTCAGGATAAAATTGTAGCTGGGTGCAAAATAGCAACTAATCTTGATTTAAGGCTTCAAAACCTCCC
GCAAGTCGGGAGGTTCGCTAAAACGCCTCGCGTTCTTAGAATACCGGATAAGCCTTCTATATCTGATTTGCTTGCTATTGGGCGCGGTAATGATTCCTACGATGAAAATAAAAACGGCTTGCTTGTTCTCGATGAGTG
CGGTACTTGGTTTAATACCCGTTCTTGGAATGATAAGGAAAGACAGCCGATTATTGATTGGTTTCTACATGCTCGTAAATTAGGATGGGATATTATTTTTCTTGTTCAGGACTTATCTATTGTTGATAAACAGGCGCG
TTCTGCATTAGCTGAACATGTTGTTTATTGTCGTCGTCTGGACAGAATTACTTTACCTTTTGTCGGTACTTTATATTCTCTTATTACTGGCTCGAAAATGCCTCTGCCTAAATTACATGTTGGCGTTGTTAAATATGGCG
ATTCTCAATTAAGCCCTACTGTTGAGCGTTGGCTTTATACTGGTAAGAATTTGTATAACGCATATGATACTAAACAGGCTTTTTCTAGTAATTATGATTCCGGTGTTTATTCTTATTTAACGCCTTATTTATCACACGGT
CGGTATTTCAAACCATTAAATTTAGGTCAGAAGATGAAATTAACTAAAATATATTTGAAAAAGTTTTCTCGCGTTCTTTGTCTTGCGATTGGATTTGCATCAGCATTTACATATAGTTATATAACCCAACCTAAGCCG
GAGGTTAAAAAGGTAGTCTCTCAGACCTATGATTTTGATAAATTCACTATTGACTCTTCTCAGCGTCTTAATCTAAGCTATCGCTATGTTTTCAAGGATTCTAAGGGAAAATTAATTAATAGCGACGATTTACAGAAG
CAAGGTTATTCACTCACATATATTGATTTATGTACTGTTTCCATTAAAAAAGGTAATTCAAATGAAATTGTTAAATGTAATTAATTTTGTTTTCTTGATGTTTGTTTCATCATCTTCTTTTGCTCAGGTAATTGAAATGA
ATAATTCGCCTCTGCGCGATTTTGTAACTTGGTATTCAAAGCAATCAGGCGAATCCGTTATTGTTTCTCCCGATGTAAAAGGTACTGTTACTGTATATTCATCTGACGTTAAACCTGAAAATCTACGCAATTTCTTTAT
TTCTGTTTTACGTGCAAATAATTTTGATATGGTAGGTTCTAACCCTTCCATTATTCAGAAGTATAATCCAAACAATCAGGATTATATTGATGAATTGCCATCATCTGATAATCAGGAATATGATGATAATTCCGCTCCT
TCTGGTGGTTTCTTTGTTCCGCAAAATGATAATGTTACTCAAACTTTTAAAATTAATAACGTTCGGGCAAAGGATTTAATACGAGTTGTCGAATTGTTTGTAAAGTCTAATACTTCTAAATCCTCAAATGTATTATCTA
TTGACGGCTCTAATCTATTAGTTGTTAGTGCTCCTAAAGATATTTTAGATAACCTTCCTCAATTCCTTTCAACTGTTGATTTGCCAACTGACCAGATATTGATTGAGGGTTTGATATTTGAGGTTCAGCAAGGTGATGC
TTTAGATTTTTCATTTGCTGCTGGCTCTCAGCGTGGCACTGTTGCAGGCGGTGTTAATACTGACCGCCTCACCTCTGTTTTATCTTCTGCTGGTGGTTCGTTCGGTATTTTTAATGGCGATGTTTTAGGGCTATCAGTTC
GCGCATTAAAGACTAATAGCCATTCAAAAATATTGTCTGTGCCACGTATTCTTACGCTTTCAGGTCAGAAGGGTTCTATCTCTGTTGGCCAGAATGTCCCTTTTATTACTGGTCGTGTGACTGGTGAATCTGCCAATGT
AAATAATCCATTTCAGACGATTGAGCGTCAAAATGTAGGTATTTCCATGAGCGTTTTTCCTGTTGCAATGGCTGGCGGTAATATTGTTCTGGATATTACCAGCAAGGCCGATAGTTTGAGTTCTTCTACTCAGGCAAG
TGATGTTATTACTAATCAAAGAAGTATTGCTACAACGGTTAATTTGCGTGATGGACAGACTCTTTTACTCGGTGGCCTCACTGATTATAAAAACACTTCTCAGGATTCTGGCGTACCGTTCCTGTCTAAAATCCCTTTA
ATCGGCCTCCTGTTTAGCTCCCGCTCTGATTCTAACGAGGAAAGCACGTTATACGTGCTCGTCAAAGCAACCATAGTACGCGCCCTGTAGCGGCGCATTAAGCGCGGCGGGTGTGGTGGTTACGCGCAGCGTGACCG
CTACACTTGCCAGCGCCCTAGCGCCCGCTCCTTTCGCTTTCTTCCCTTCCTTTCTCGCCACGTTCGCCGGCTTTCCCCGTCAAGCTCTAAATCGGGGGCTCCCTTTAGGGTTCCGATTTAGTGCTTTACGGCACCTCGAC
CCCAAAAAACTTGATTTGGGTGATGGTTCACGTAGTGGGCCATCGCCCTGATAGACGGTTTTTCGCCCTTTGACGTTGGAGTCCACGTTCTTTAATAGTGGACTCTTGTTCCAAACTGGAACAACACTCAACCCTATC
TCGGGCTATTCTTTTGATTTATAAGGGATTTTGCCGATTTCGGAACCACCATCAAACAGGATTTTCGCCTGCTGGGGCAAACCAGCGTGGACCGCTTGCTGCAACTCTCTCAGGGCCAGGCGGTGAAGGGCAATCAG
CTGTTGCCCGTCTCACTGGTGAAAAGAAAAACCACCCTGGCGCCCAATACGCAAACCGCCTCTCCCCGCGCGTTGGCCGATTCATTAATGCAGCTGGCACGACAGGTTTCCCGACTGGAAAGCGGGCAGTGAGCGC
AACGCAATTAATGTGAGTTAGCTCACTCATTAGGCACCCCAGGCTTTACACTTTATGCTTCCGGCTCGTATGTTGTGTGGAATTGTGAGCGGATAACAATTTCACACAGGAAACAGCTATGACCATGATTACGAATTC
GAGCTCGGTACCCGGGGATCCTCTAGAGTCGACCTGCAGGCATGCAAGCTTGGCACTGGCCGTCGTTTTACAACGTCGTGACTGGGAAAACCCTGGCGTTACCCAACTTAATCGCCTTGCAGCACATCCCCCTTTCG
CCAGCTGGCGTAATAGCGAAGAGGCCCGCACCGATCGCCCTTCCCAACAGTTGCGCAGCCTGAATGGCGAATGGCGCTTTGCCTGGTTTCCGGCACCAGAAGCGGTGCCGGAAAGCTGGCTGGAGTGCGATCTTCC
TGAGGCCGATACTGTCGTCGTCCCCTCAAACTGGCAGATGCACGGTTACGATGCGCCCATCTACACCAACGTGACCTATCCCATTACGGTCAATCCGCCGTTTGTTCCCACGGAGAATCCGACGGGTTGTTACTCGCT
CACATTTAATGTTGATGAAAGCTGGCTACAGGAAGGCCAGACGCGAATTATTTTTGATGGCGTTCCTATTGGTTAAAAAATGAGCTGATTTAACAAAAATTTAATGCGAATTTTAACAAAATATTAACGTTTACAAT
TTAAATATTTGCTTATACAATCTTCCTGTTTTTGGGGCTTTTCTGATTATCAACCGGGGTACATATGATTGACATGCTAGTTTTACGATTACCGTTCATCGATTCTCTTGTTTGCTCCAGACTCTCAGGCAATGACCTGA
TAGCCTTTGTAGATCTCTCAAAAATAGCTACCCTCTCCGGCATTAATTTATCAGCTAGAACGGTTGAATATCATATTGATGGTGATTTGACTGTCTCCGGCCTTTCTCACCCTTTTGAATCTTTACCTACACATTACTC
AGGCATTGCATTTAAAATATATGAGGGTTCTAAAAATTTTTATCCTTGCGTTGAAATAAAGGCTTCTCCCGCAAAAGTATTACAGGGTCATAATGTTTTTGGTACAACCGATTTAGCTTTATGCTCTGAGGCTTTATTG
CTTAATTTTGCTAATTCTTTGCCTTGCCTGTATGATTTATTGGATGTT

Supplementary Figure 13. Sequence of M13mp18 ssDNA

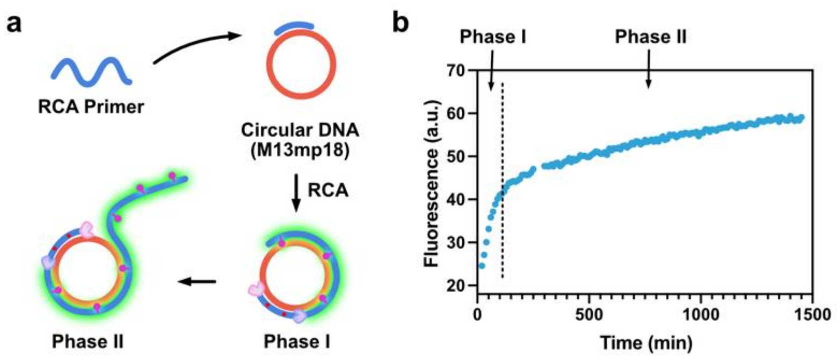

Supplementary Figure 14. RCA amplification and in situ labeling Fluorescence amplification with 100% dUTP was observed to constitute two phases in which the fluorescence enhancement was initially rapid, followed by a slow further increase in the second phase. We hypothesize that the in situ RCA and labeling afforded circular dsDNA, providing a rigid environment for the incorporated dyes, while the slower phase may reflect strand displacement to extend the product strand further. Given that the longest stretch of consecutive adenines in M13mp18 ssDNA is only six consecutive adenines, it leaves the question as to why the optimized ratio is lower than one dUTP molecule out of 50 nucleotides, not one out of 6 nucleotides (Figure 4a). We attributed this to the favored dUTP incorporation by Klenow enzyme (Kf). Considering that 25% and 50% dUTP in the mix resulted in ~50% and ~75% incorporation of dU, respectively, Kf Pol seems to incorporate dUTP approximately three times more favorably than dTTP. According to this back-of-the-envelope calculation, the ratio of dUTP should be lower than one dUTP molecule out of 18 nucleotides to pass through a six consecutive adenine sites with one labeling, which is consistent with the observations in Figure 4a. The fluorescence was measured on a Fluoroskan Ascent microplate reader (485 nm/538 nm) at 37℃.

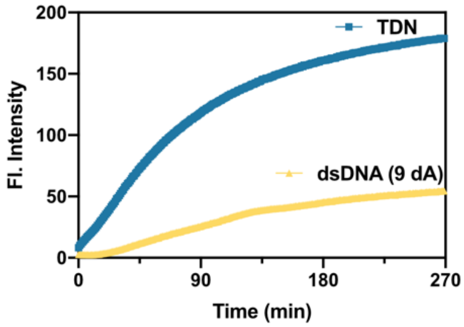

Supplementary Figure 15. Fluorescence enhancement during BETr labeling with a tetrahedral DNA nanostructure (TD) and dsDNA (**9dA**) showing that TD containing six CCVJ-1 is even brighter than **9dA** containing nine CCVJ-1, plausibly due to a ridic duplex context. TD was prepared by annealing ssDNA-1, ssDNA-2, ssDNA-3, and ssDNA-linker (2 µM each) by annealing it for 3 min at 95 ℃. dsDNA was prepared with **9dA** (2 µM), 10 µM primer, 50 µM of dNTP (dA, dG, dC), 100 µM dUTP, and 2 U/mL of Klenow. BETr labeling proceeded with 20 µM CCVJ-1 and 20 U/mL UDG. The fluorescence was measured on a Fluoroskan Ascent microplate reader (485 nm/538 nm) at 37℃.

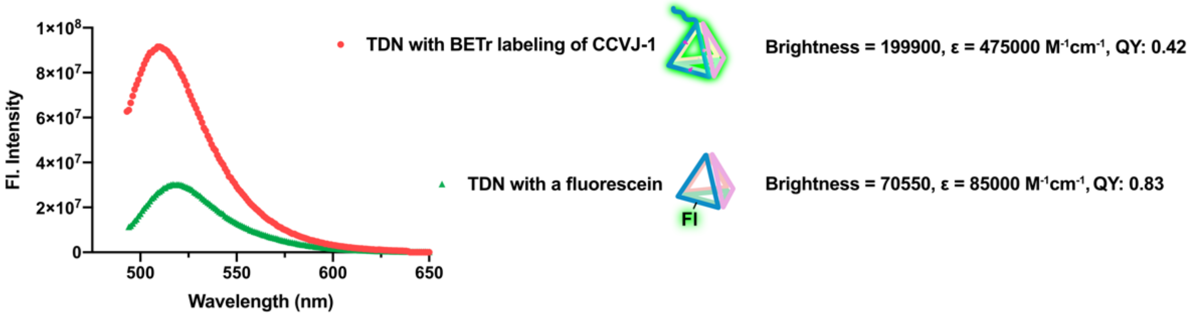

Supplementary Figure 16. Fluorescence intensity comparison of TD components labeled with CCVJ-1 through BETr and fluorescein through solid phase synthesis TD was prepared with ssDNA-1, ssDNA-2, ssDNA-3, and ssDNA-linker (2 µM each) by annealing for 3 min at 95°C. BETr labeling was performed with 20 µM CCVJ-1 and 20 U/mL UDG. Photophysical properties were calculated based on the reported value of fluorescein in dsDNA.[5] The fluorescence was measured on a Jobin Yvon-Spex Spectrometer at 37°C.

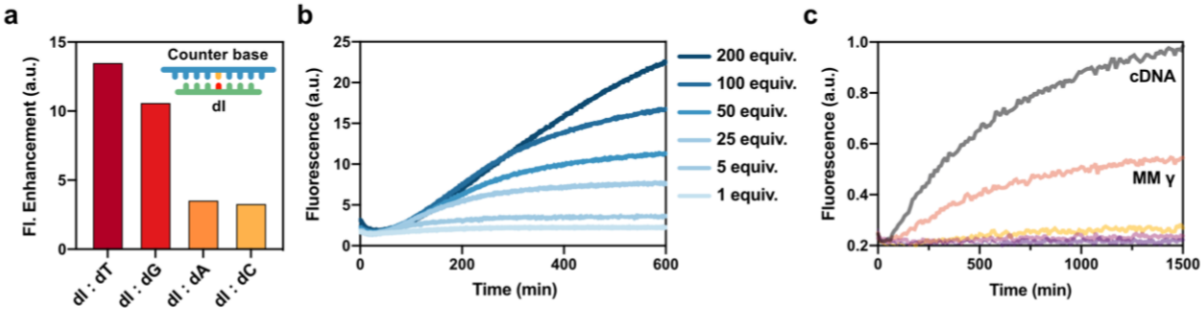

Supplementary Figure 17. Isothermal turnover detection (a) Fluorescence enhancement comparison depending on the counter base of the deaminated base (dI in 11mer) to be repaired. (b) Fluorescence responses while detecting target DNA (Template dT) with 11mer depending on the relative amount of probe oligo and target DNA. (c) Fluorescence intensity change over time upon the detection of target DNAs containing single mismatch in varied positions. [Target DNA] = 2 µM (a,c), 200 nM (b), [Probe oligo] = 2 µM (a,c), [UBER] = 10 µM (a,c), 100 µM in (b), [MPG] = 100 U/mL. The fluorescence was measured on a Fluoroskan Ascent microplate reader (485 nm/538 nm) at 37°C. The bar graphs were plotted with the data at 600 min of incubation.

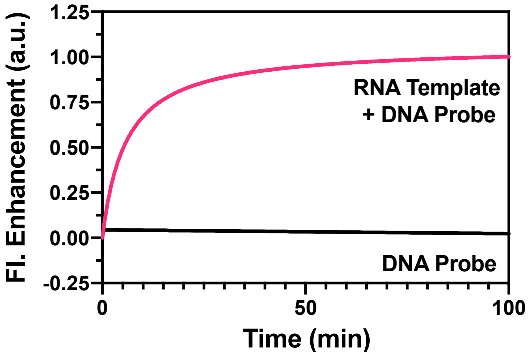

Supplementary Figure 18. Target RNA detection with turnover labeling Application of BETr labeling with turnover on an RNA template. Normalized fluorescence enhancement of isothermal turnover detection with Target RNA and probe DNA (11 mer). [Target RNA] = 10 μM, [Probe DNA] = 10 μM, [UBER] = 10 μM, [MPG] = 50 U/mL. A smoothing function was applied. The fluorescence intensities were measured on a Fluoroskan Ascent microplate reader (485 nm/538 nm) at 37 °C.

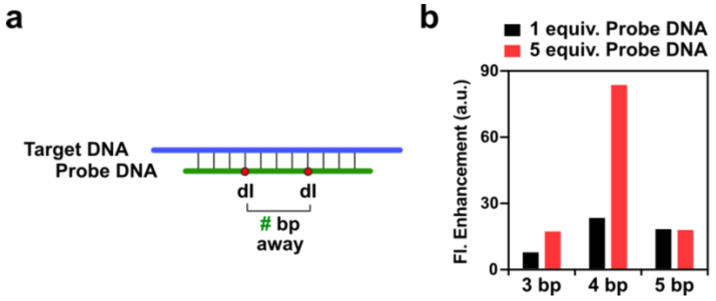

Supplementary Figure 19. Isothermal turnover detection with longer DNA probes with two deoxyinosines at different positions (a) Illustration of longer probe DNA design with multiple dI. (b) Fluorescence enhancement under the BETr labeling condition with 1 equivalent of target DNA (3bp, 4bp, 5bp) in the presence of 1 or 5 equivalent of probe DNA strand. [Long Target DNA] = 2 μM, [Probe DNA] = 2 or 10 μM, [UBER] = 20 μM, [MPG] = 50 U/mL. The fluorescence intensities were measured on a Fluoroskan Ascent microplate reader (485 nm/538 nm).

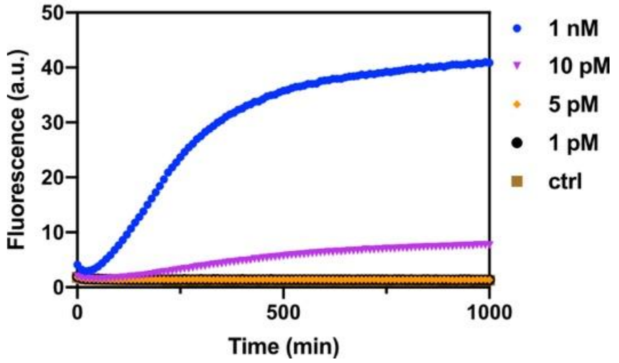

Supplementary Figure 20. **Measurement of the detection limit of the isothermal turnover detection system** Varied concentration of target DNA (**Template dT**) was labeled with 10 μM of Probe DNA (**11mer**), 100 U/mL MPG, and 100 μM of UBER in Buffer B (Supplementary Figure 6) with a total volume of 50 μL. The fluorescence was measured on a Fluoroskan Ascent microplate reader (485 nm/538 nm) at 37 °C.

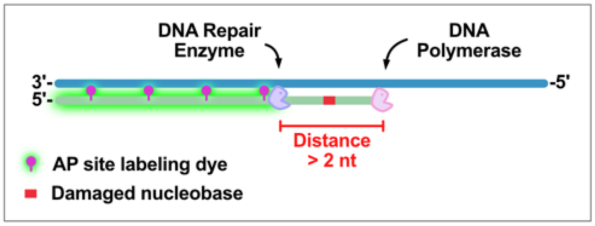

Supplementary Figure 21. We further investigated the mechanism of the *in situ* labeling machinery regarding the relative positioning between the polymerase and the glycosylase activities. Given that the kinetics of the glycosylase was observed to be much faster than that of the Kf polymerase (Fig. 1c,d), we anticipated that the uracil DNA lesion is likely excised shortly after it is incorporated. The question here is how close to the polymerase is the incorporated dU available for the repair by the glycosylase. As the number of flanking base pairs alongside a lesion significantly affects the activity of glycosylases,[6-7] the DNA excision and labeling efficiency was tested with DNAs having uracil near the 3′-end with varied distance from the end (Fig. 2d). The fluorescence intensity with more flanking base pairs at the end of dsDNA exhibited stronger fluorescence intensities. We surmise that more flanking base pairs in dsDNA provided a more rigid environment to fluorophores, inducing higher fluorescence intensity. The results imply that after the incorporation of a DNA lesion by a polymerase, the following glycosylase holds until the polymerase synthesizes at least two more bases after the lesion, then the lesion is repaired and labeled.

Uncropped electrophoresis gels presented in supplementary figures.

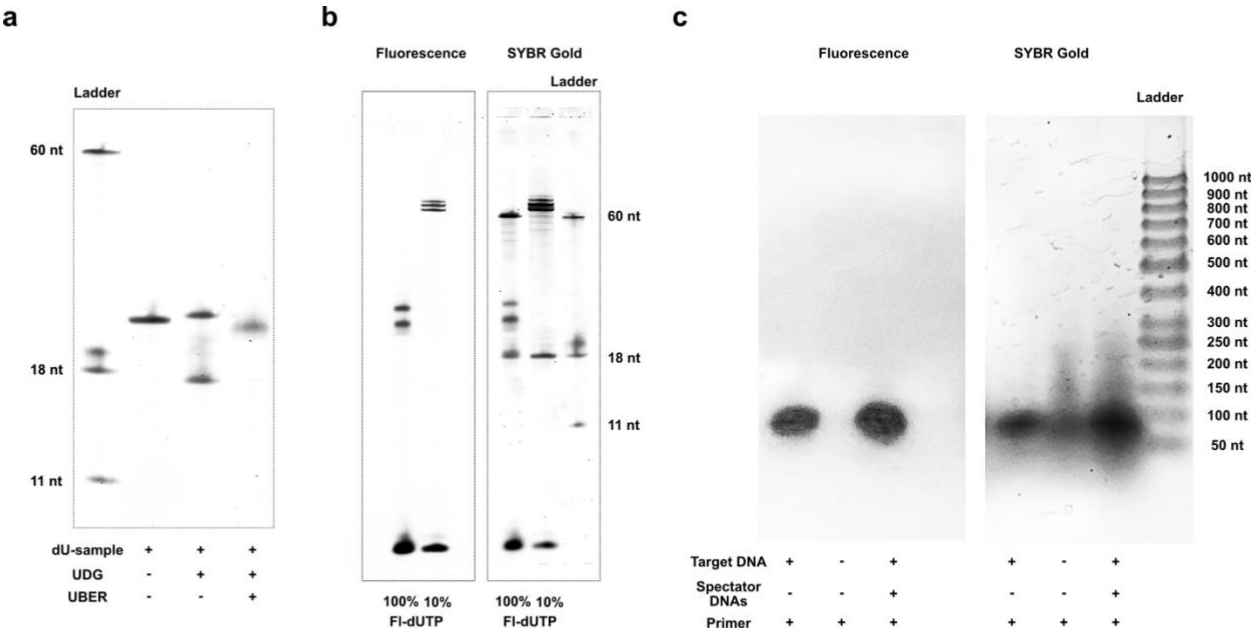

a: Supplementary Figure 4, **b:** Supplementary Figure 8d, and **c:** Figure 3f.

References

1. Wilson, D. L.; Kool, E. T., Ultrafast Oxime Formation Enables Efficient Fluorescence Light-Up Measurement of DNA Base Excision. *J. Am. Chem. Soc.* **2019,** *141* (49), 19379–19388.
2. Hui, J. Z.; Tamsen, S.; Song, Y.; Tsourkas, A., LASIC: Light Activated Site-Specific Conjugation of Native IgGs. *Bioconjug. Chem.* **2015,** *26* (8), 1456–1460.
3. Javorskis, T.; Sriubaitė, S.; Bagdžiūnas, G.; Orentas, E., N-Protected 1, 2-Oxazetidines as a Source of Electrophilic Oxygen: Straightforward Access to Benzomorpholines and Related Heterocycles by Using a Reactive Tether. *Chem. Eur. J.* **2015,** *21* (25), 9157–9164.
4. Hui, J. Z.; Tamsen, S.; Song, Y.; Tsourkas, A., LASIC: Light Activated Site-Specific Conjugation of Native IgGs. *Biocong. Chem.* **2015,** *26* (8), 1456–1460.
5. Delgadillo, R. F.; Parkhurst, L. J., Spectroscopic Properties of Fluorescein and Rhodamine Dyes Attached to DNA. *Photochem. Photobiol.* **2010,** *86* (2), 261–272.
6. Edwards, S. K.; Ono, T.; Wang, S.; Jiang, W.; Franzini, R. M.; Jung, J. W.; Chan, K. M.; Kool, E. T., In Vitro Fluorogenic Real-Time Assay of the Repair of Oxidative DNA Damage. *ChemBioChem* **2015,** *16* (11), 1637.
7. Jun, Y. W.; Wilson, D. L.; Kietrys, A. M.; Lotsof, E. R.; Conlon, S. G.; David, S. S.; Kool, E. T., An Excimer Clamp for Measuring Damaged-Base Excision by the DNA Repair Enzyme NTH1. *Angewandte Chemie* **2020,** *132* (19), 7520–7525.
