# Supplementary material for: Efficient DNA fluorescence labeling via base excision trapping
Source: Nat Commun. 2022 Aug 26;13:5043. doi: 10.1038/s41467-022-32494-8 (PMC9418136; doi:10.1038/s41467-022-32494-8)
Supplement: Supplementary file 2 — Reporting Summary [file 41467_2022_32494_MOESM2_ESM.pdf]

nature portfolio

Corresponding author(s): Eric T. Kool

Last updated by author(s): 7/29/2022

Reporting Summary

Nature Portfolio wishes to improve the reproducibility of the work that we publish. This form provides structure for consistency and transparency in reporting. For further information on Nature Portfolio policies, see our Editorial Policies and the Editorial Policy Checklist.

Statistics

For all statistical analyses, confirm that the following items are present in the figure legend, table legend, main text, or Methods section.

n/a	Confirmed	
☐	☒	The exact sample size (*n*) for each experimental group/condition, given as a discrete number and unit of measurement
☐	☒	A statement on whether measurements were taken from distinct samples or whether the same sample was measured repeatedly
☒	☐	The statistical test(s) used AND whether they are one- or two-sided<br>*Only common tests should be described solely by name; describe more complex techniques in the Methods section.*
☒	☐	A description of all covariates tested
☐	☒	A description of any assumptions or corrections, such as tests of normality and adjustment for multiple comparisons
☒	☐	A full description of the statistical parameters including central tendency (e.g. means) or other basic estimates (e.g. regression coefficient) AND variation (e.g. standard deviation) or associated estimates of uncertainty (e.g. confidence intervals)
☒	☐	For null hypothesis testing, the test statistic (e.g. *F*, *t*, *r*) with confidence intervals, effect sizes, degrees of freedom and *P* value noted<br>*Give P values as exact values whenever suitable.*
☒	☐	For Bayesian analysis, information on the choice of priors and Markov chain Monte Carlo settings
☒	☐	For hierarchical and complex designs, identification of the appropriate level for tests and full reporting of outcomes
☒	☐	Estimates of effect sizes (e.g. Cohen's *d*, Pearson's *r*), indicating how they were calculated

Our web collection on statistics for biologists contains articles on many of the points above.

Software and code

Policy information about availability of computer code

Data collection	The data was collected with Prism, and microsoft excel. The figures were drawn in Affinity Designer
Data analysis	The fluorescence images were processed in image J,

For manuscripts utilizing custom algorithms or software that are central to the research but not yet described in published literature, software must be made available to editors and reviewers. We strongly encourage code deposition in a community repository (e.g. GitHub). See the Nature Portfolio guidelines for submitting code & software for further information.

Data

Policy information about availability of data

All manuscripts must include a data availability statement. This statement should provide the following information, where applicable:

- Accession codes, unique identifiers, or web links for publicly available datasets
- A description of any restrictions on data availability
- For clinical datasets or third party data, please ensure that the statement adheres to our policy

The data that support the findings of this study is in Figshare (https://doi.org/10.6084/m9.figshare.20398704.v1).

Human research participants

Policy information about studies involving human research participants and Sex and Gender in Research.

Reporting on sex and gender	N/A
Population characteristics	N/A
Recruitment	N/A
Ethics oversight	N/A

Note that full information on the approval of the study protocol must also be provided in the manuscript.

Field-specific reporting

Please select the one below that is the best fit for your research. If you are not sure, read the appropriate sections before making your selection.

- [x] Life sciences
- [ ] Behavioural & social sciences
- [ ] Ecological, evolutionary & environmental sciences

For a reference copy of the document with all sections, see nature.com/documents/nr-reporting-summary-flat.pdf

Life sciences study design

All studies must disclose on these points even when the disclosure is negative.

Sample size	In vitro labeling is highly reproducible chemical reaction as shown in Supplementary Figure 2, therefore the sample size is (n = 1) in these cases. Gel analyses have been repeated at least three times and showed the identical results.
Data exclusions	No data were excluded
Replication	All replication were highly successful (Supplementary Figure 2)
Randomization	Randomization was not needed in this research, as this work was designed for the labeling at designated sites
Blinding	Blinding was not needed in this research, as this work was designed for the labeling at designated sites

Reporting for specific materials, systems and methods

We require information from authors about some types of materials, experimental systems and methods used in many studies. Here, indicate whether each material, system or method listed is relevant to your study. If you are not sure if a list item applies to your research, read the appropriate section before selecting a response.

Materials & experimental systems

n/a	Involved in the study	
☐	☒	Antibodies
☐	☒	Eukaryotic cell lines
☒	☐	Palaeontology and archaeology
☒	☐	Animals and other organisms
☒	☐	Clinical data
☒	☐	Dual use research of concern

Methods

n/a	Involved in the study	
☒	☐	ChIP-seq
☒	☐	Flow cytometry
☒	☐	MRI-based neuroimaging

Antibodies

Antibodies used	Primary antibody anti-G3BP-1, Sigma-Aldrich, SAB4500043, LOT: 217089, Dilution (1:500)
Validation	Sigma-Aldrich has validated the antibody for ELISA ans Western blotting (https://www.sigmaaldrich.com/US/en/coa/SIGMA/SAB4500043/217089)

Eukaryotic cell lines

Policy information about cell lines and Sex and Gender in Research

Cell line source(s)	HeLa cell purchased from ATCC, HEK293T purchased from ATCC
Authentication	Both cell lines were not authenticated further.
Mycoplasma contamination	Confirmed that the cell line tested negative for mycoplasma contamination.
Commonly misidentified lines (See ICLAC register)	Both are no commonly misidentified cell lines.
